# Green synthesis and characterization of zirconium nanoparticlefor dental implant applications

**DOI:** 10.1016/j.heliyon.2022.e12711

**Published:** 2022-12-30

**Authors:** Mohammad Asaduzzaman Chowdhury, Nayem Hossain, Md. Golam Mostofa, Md. Riyad Mia, Md. Tushar, Md. Masud Rana, Md. Helal Hossain

**Affiliations:** a Department of Mechanical Engineering, Dhaka University of Engineering and Technology (DUET), Gazipur, Gazipur, 1707, Bangladesh; b Department of Mechanical Engineering, IUBAT-International University of Business Agriculture and Technology, Bangladesh; c Department of Mechanical Engineering, Dhaka University of Engineering and Technology, Gazipur, Bangladesh

**Keywords:** Zirconium manoparticles, Green synthesis, Antimicrobial activity, Morphological analysis, Dental implant applications

## Abstract

Green synthesis is a promising and cost-effective technique to synthesize nanoparticles from plant extract. The present study shows the green synthesis of zirconium nanoparticles using the extract of ginger, garlic, and zirconium nitride. The obtained nanoparticles were studied for potential dental implant applications. The synthesized nanoparticles were characterized by Fourier Transform Infrared Spectroscopy (FTIR), Field Emission Scanning Electron Microscopy (FESEM), Energy Dispersive X-Ray Spectroscopy (EDX), X-Ray diffraction analysis (XRD), and antibacterial analysis. FTIR analysis confirmed the presence of various organic compounds in the synthesized nanoparticles. The synthesized nanoparticles were spherical, triangular, and irregular, with varying sizes confirmed by FESEM analysis. The nanoparticles synthesized from the combination of garlic and ginger, and zirconium exhibited potent antibacterial activity against *S. aureus*. Anti-biofilm, anti-microbial activity, biointegration formation, and cell mechanism survival are also mentioned. Thus, the synthesized nanoparticles can be a good candidate for a dental implant because of their excellent antimicrobial properties.

## 1 Introduction

Nanotechnology and nanoscience are the study and application of extremely small objects with dimensions ranging from one to one hundred micrometers. Nanotechnology is being used in fields such as chemistry, biology, physics, materials science, and engineering [1]. Nanoparticle synthesis the term to refers the process of making nanoparticles by combing two or more ingredients into one [2,3]. Nanoparticles exhibit unique physical, chemical, and green features that are not found in bulk materials [4]. This unique physicochemical property, along with other specific features of metallic nanoparticles such as small size, high surface to volume ratio, surface charge, and surface chemistry, provide a significant opportunity to design diagnostic agents for diagnostic purposes and targeted drug delivery systems, in addition to benefiting from the intrinsic pharmaceutical potential of these nanoparticles [[5], [6], [7]]. Nanoparticles can be synthesized by chemical and green synthesis whereas chemical synthesis of nanoparticles requires toxic chemicals, high energy, and the cost is high. On the other hand, the green synthesis of nanoparticles requires less energy and costs less money [8].

Zirconium is a transition metal that is essentially identical to titanium in terms of corrosion resistance [9]. Zirconium dental crowns and biomaterials are frequently employed [10]: [11]. Zirconium nanoparticles are also used for biosensors, anticancer, antimicrobials, antioxidants, and implants [12]. Drug delivery carriers for a variety of medications, including penicillin, alendronate, itraconazole, and others, have also effectively used zirconium nanoparticles [13]. Ag nanoparticles are also synthesized using green synthesis method. Besides, Ag nanoparticles can be synthesized along with Zr nanoparticles as hybrid nanoparticles [[14], [15], [16], [17]].

The rhizome or root of the ginger plant (ZingiberOfficinale) is widely used as a flavoring and herbal medicine [18]. It's also high in antioxidants, which protect DNA from stress and damage in the human body. They may help to avoid chronic diseases like hypertension, heart disease, and lung disease, as well as promote healthy aging. Ginger's active ingredients have also been linked to the prevention of cancers such as gastrointestinal cancer, pancreatic cancer, liver cancer, skin cancer, and others [19,20].

Garlic (Allium sativum) is a plant in the onion family [21]. Garlic can help lower cholesterol and blood pressure levels. It contains cancer-prevention chemicals that protect cells from damage and maturation. Allicin, a component of garlic, has antibacterial, antifungal, antiviral, and antiseptic properties. Allicin aids in the elimination of microorganisms that cause acne [22]. It also helps with edema and inflammation reduction, as well as blood circulation enhancement. The skin is able to absorb more nutrients as a result of these beneficial impacts.

This study to aim progress of “Green Synthesis of Zirconium Nanoparticles by Using Ginger and Garlic Extract” used for promising nanocarriers for drug delivery in biomedical applications. In this research Zirconium nitrate ZrN, Ginger, and Garlic are used. Zirconium nitrate ZrNis a volatile anhydrous and highly water-soluble crystalline transition metal nitrate salt of Zirconium. The phytochemical analysis in Ginger and Garlic water extract showed a high presence of flavonoids and phenolics components, which are essential in the metal nanoparticle synthesis reduction method. The Zirconium nanoparticles prepared with this extract by using the synthesis reduction method, it's indicating that this extract is able to green bioreduction to Zirconium nanoparticles. These region nanoparticles were found to be extremely stable.

Dental implants are a dependable and well-documented therapeutic option for partially or totally edentulous patients. For decades, titanium has been the gold standard for dental implants, with great long-term survival and success rates. Despite their excellent biomechanical qualities, titanium implants appear to have certain drawbacks, including probable peri-implant mucosa discoloration, and hypersensitivity reactions, and contribute to the development of peri-implantitis [23].

Zirconium has been proposed as a substitute for titanium implants. The tooth-matched color may be able to overcome titanium's possible drawbacks, especially in the cosmetic areas of thin soft tissue biotypes. Furthermore, compared to titanium, zirconium is biocompatible and has a lower risk of early plaque formation. In comparison to Ti, Zr-based implants have demonstrated promising clinical results with low ion release, lesser cytotoxicity, good biocompatibility, excellent strength, fracture toughness, and good osseointegration ability [24]. Zirconium is a transition metal with excellent corrosion resistance as well as improved mechanical, thermal, catalytic, and mechanical properties. As a result, zirconium has been employed to make implant biomaterials and dental crowns.

Many studies have shown the use of zirconium in a variety of applications, including adsorption, photo-degradation, antibacterial agents, and structural reinforcement. Various research has reported on the use of ZrNPs as biosensors, anticancer agents, antibacterial agents, antioxidants, and implants.

The novelty of this study is to synthesize zirconium nitride in a nanoscale in combination with ginger and garlic extracts in order to overcome the limitations and constraints of traditional dental materials. Titanium exhibited great biocompatibility, corrosion resistance, and acceptable mechanical qualities, which were especially important during the early stages of osseointegration. Despite this, titanium implant failures were difficult to avoid. Several ways have been explored to improve titanium surfaces, one of which was to deposit a coating on the surface of titanium [25]. In comparison to other hard coatings, such as TiN, ZnN coating has better thermal stability and corrosion resistance at high temperatures, and ZrN coatings have the potential to be used as a biocompatible material as well [26]. Zirconium nitride is in the form of nanoparticles used as a potential coating material to help the titanium implants osseointegrate better (i). The unique qualities of zirconia, such as enhanced toughness, strength, fatigue resistance, and corrosion resistance, made it an attractive implant material as humans went toward greater exploration (ii). Furthermore, once the buildup of titanium particles was discovered in tissues near the implants and nearby lymph nodes, physicians and patients began to question the safety of titanium implants (iii). Some people have complained of allergies caused by titanium implants (iv). There is currently no information on the toxicity of zirconia implants. Nanoparticles have proven to be more efficient than traditional materials in terms of bonding and surface chemistry (v). These bio-nanoparticles have been integrated into many dental materials for their antibacterial activities and have aided in the treatment of oral illnesses, and eradication of smear layer and biofilms [27] (vi). These nanoparticles will bring a new paradigm shift in dentistry by combining all of their benefits. Nanoparticles (NPs) are a breakthrough in the treatment and prevention of dental infections. Because of their positive charge and larger surface area, NPs can react with negatively charged bacterial cells, resulting in greater antibacterial action. NPs can also be mixed with polymers or deposited onto biomaterial surfaces. This was also proven to have improved antibacterial properties. Nanomaterials have shown potential in reducing biofilm development, enhancing remineralization of tooth structure by limiting demineralization, and combating caries-related and endodontic bacteria. When compared to bulk materials, changes in specific properties such as size, shape, and surface area increase the green activity of nanoparticles [28].

## 2 Methodology

Extraction (garlic, ginger), creation of an aqueous reagent solution, synthesis of reagent and extract, centrifugation, drying, powdering, and other processes or steps were used in this study.

### 2.1 Materials

#### 2.1.1 Extraction process (garlic, ginger)

**Fig. 1:**
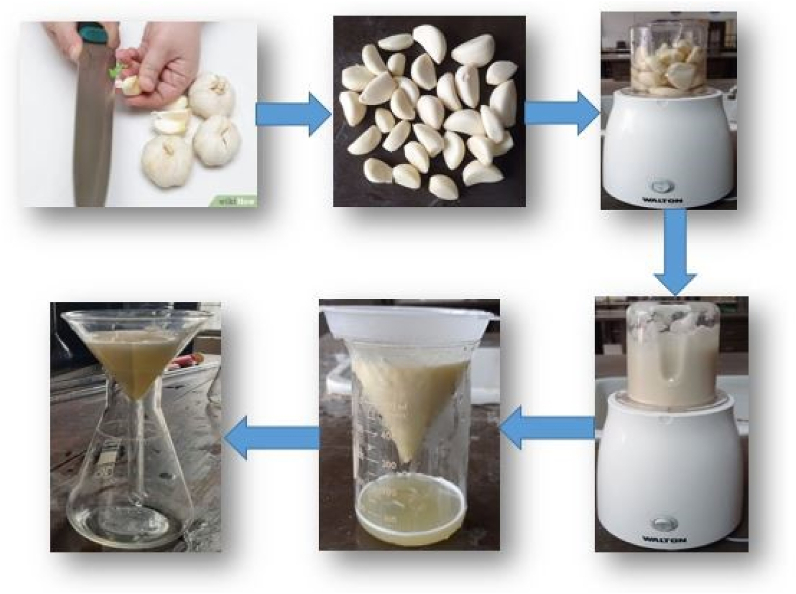
Extraction process of garlic.

Both Garlic and Ginger were bought from the local market. Then peeled the garlic bulbs and ginger rhizomes and properly washed by using distilled water. After washing, garlic and ginger were cut into small pieces for the next purpose. The amount of 100 g of garlic bulbs was taken with 100 ml distilled water and ground this mixture using a blender. The blended mixture was filtrated by using a cloth to a reasonable liquid state. Finally, this liquid was filtrated by Whatman-1 filter paper for getting pure extract without any tiny particles of garlic blub. Fig. 1 shows the extraction process of garlic.

In a similar way, ginger rhizomes extracted were also prepared. In this research work, four observations were performed using three types of extract. These three extract are garlic, ginger, and a combination of garlic, and ginger extract. The combination of garlic and ginger extract is 50 g Ginger +50 g Garlic with 100 ml distilled water. Then this combined mixture was ground using the blender. And the rest of the steps of extraction were performed according to the above.

#### 2.1.2 Preparation of aqueous solution of ${ZrO\left({NO}_{3}\right)}_{2}$

**Fig. 2:**
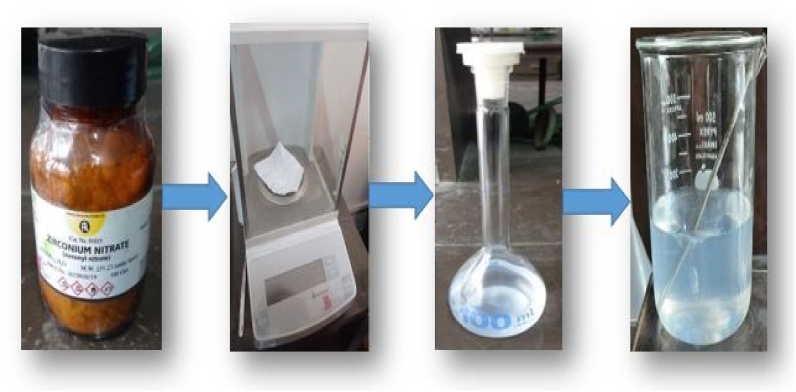
Steps of making the aqueous solution of ${ZrO\left({NO}_{3}\right)}_{2}\text{.}$

Using distilled water, the aqueous solution reagent was created. This solution was made in a 250 ml solution with a concentration of 1 cM (centi-molar). Hydrous (1 molecule H_2_O) has a molecular weight of 249.224 g. 0.625 gm and 250 ml distilled water were combined in a volumetric flask to make a 1 cM 250 ml solution. The solution was then carefully agitated to obtain a saturated mixing of water and reagent in the flask. Finally, a 500 ml beaker was filled with this solution shown in Fig. 2.

#### 2.1.3 Synthesis process of reagent solution with extract (garlic, ginger)

**Fig. 3:**
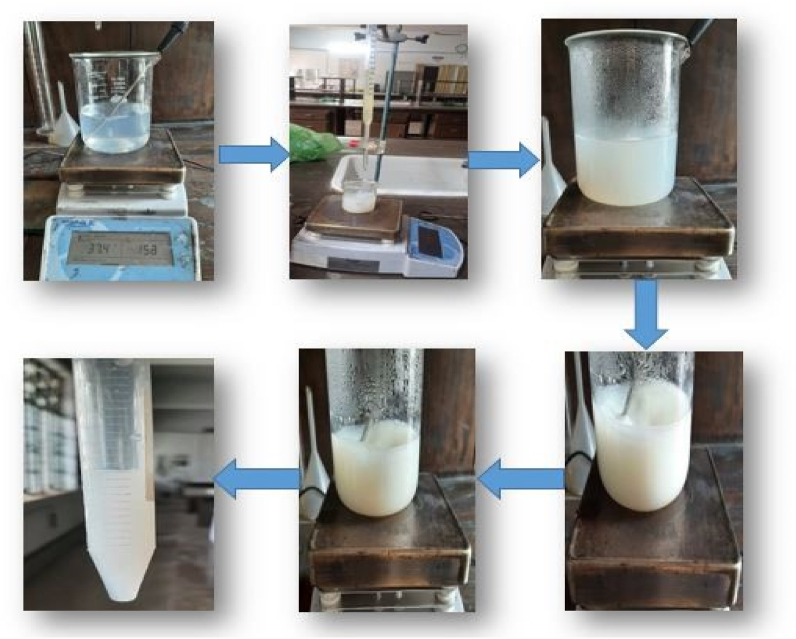
Steps of synthesis process.

First the aqueous solution of 250 ml was taken into a 500 ml beaker. Then 50 ml garlic or ginger extract was poured into a burette by using a pipette with the burette stand. Then the beaker was placed on the magnetic stirrer set at 300 rpm, heating 80–90 temperature at 2.5 h. The extract from the burette was gradually dropwise added into the aqueous solution to maintain a 5:1 ratio of the aqueous solution and extract. After completing this process the color of the mixture was changed from light blue to white and the solution amount was decreased. This hot synthesis mixture was cooled at normal temperature. After cooling, the synthesis solution was poured into falcon tubes for the next purpose. Fig. 3 shows the steps of the synthesis process.

Similarly, the ginger extract with an aqueous solution of the synthesis solution was prepared according to the above process. And garlic and ginger combination extract 50 ml (1:1) of the combined extract was taken into the burette and the next steps were the same as in the above process.

#### 2.1.4 Centrifuge process

The falcon tubes were filled with the cooled synthesis solution. The falcon tubes carrying the synthesis solution were inserted into the centrifuge machine while preserving weight balance for centrifugation. After that, centrifuge at 5000 rpm for 20 min. The nanoparticles were precipitated beneath the falcon tube after centrifugation. The water that was above the precipitated particles was removed. The precipitate was then moved on to the next stage of drying.

**Fig. 4:**
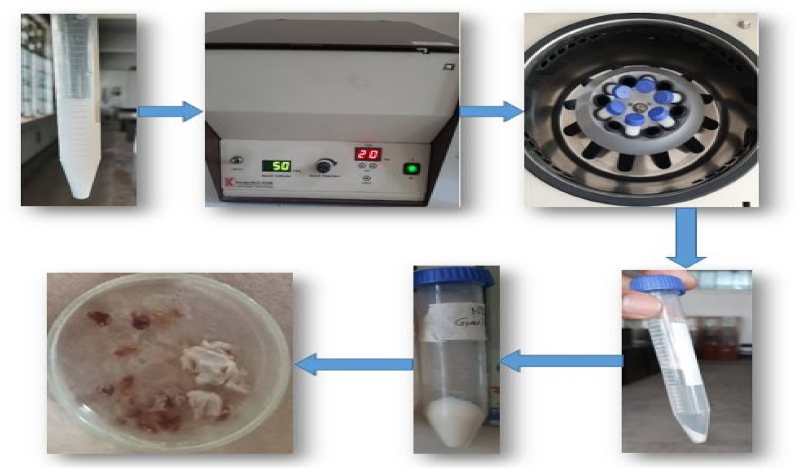
Steps of the centrifuge process.

All four observations were centrifuged in the same method. Among the four observations, there does no need for synthesis with organic extract (garlic, ginger) in the case of zirconium. Here firstly, the aqueous solution of zirconium nitrate 1 cM 250 ml was prepared according to the previously mentioned steps. The aqueous solution was stirred and heated using the magnetic stirrer set 80–90deg centigrade temperature 300 rpm for 2.5 h. Naturally cooled this aqueous solution and poured it into falcon tubes for centrifuging then drying and so on shown in Fig. 4.

#### 2.1.5 Drying process

Using a spatula and a lustrous glass rod, the precipitate, paste form, and produced nanoparticles were removed from the falcon tube and placed on a Petri plate. The Petri dish containing paste from nanoparticles was then dried in the open air and exposed to sunshine. A filter or thin cloth was placed over the Petri dish to ensure that it was free of dust and other undesirable particles.

### 2.2 Characterization

#### 2.2.1 FTIR analysis

FTIR analysis was accomplished within the frequency range from 500 cm^−1^ to 4000 cm^−1^ by PerkinElmer, USA-made spectrometer.

#### 2.2.2 Surface morphology analysis

The surface of the synthesized nanoparticles was analyzed by the FESEM-EDX made by JEOL, Japan, at different resolutions.

#### 2.2.3 XRD analysis

Further analysis of the synthesized nanoparticles was performed using a Bruker D8 advanced X-ray beam diffraction analyzer, Germany to find the crystal structure.

#### 2.2.4 Antimicrobial analysis

The antibacterial properties of the synthesized nanoparticles were characterized by the Kirby-Bauer disk diffusion test method maintaining ASTM E2149-01 standard where the concentration of bacterial cells was 1000 CFU/ml and the concentration of nanoparticles was 200 mg/ml. The test was conducted against both gram-positive and gram-negative bacteria to find the usability of the nanoparticles in dental implant applications.

#### 2.2.5 Cytotoxicity analysis

The cytotoxicity of the synthesized nanoparticles was performed using the Vero cell line in WAFFEN Laboratory, Dhaka, Bangladesh.

## 3 Result and discussion

Utilizing Ginger and Garlic Extract, zirconium nanoparticles were produced using a green approach. The effects of several parameters, such as stirring temperature, Ginger and Garlic extract concentration, and calculating temperature, were investigated, and conditions for the synthesis of Zirconium nanoparticles were adjusted.

### 3.1 FTIR analysis

**Fig. 5:**
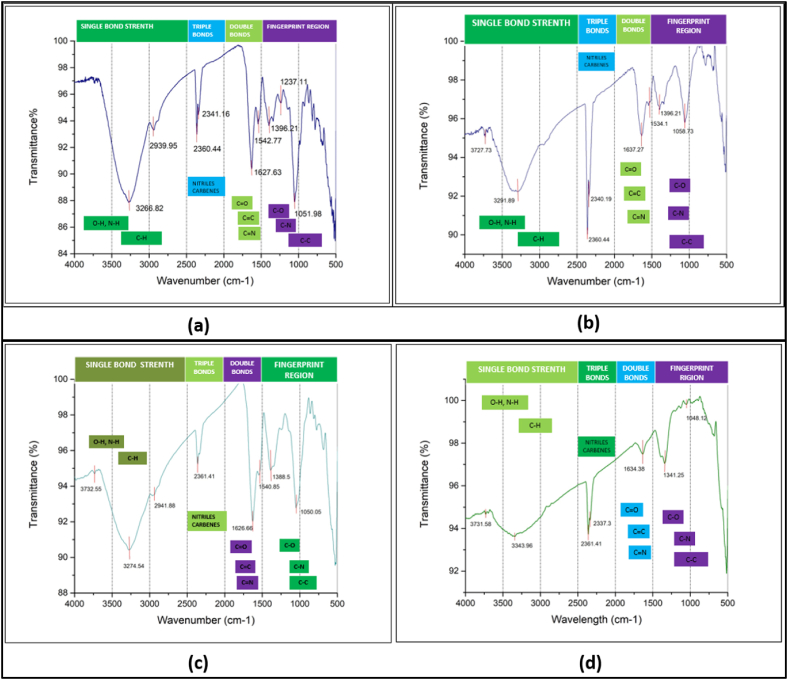
FTIR analysis of the synthesized (a) Garlic added ZrNnanoparticle, (b) Ginger added ZrN nanoparticle, (c) Garlic and Ginger added ZrN nanoparticle (d) ZrN nanoparticle.

The FTIR spectra of the synthesized zirconium nanoparticles were represented in Fig. 5. The spectrum of the nanoparticle synthesized from Garlic (Fig. 5a) shows a broad spectrum at 3266 cm^−1^ which is attributed to the strong stretching of carboxylic acid O–H. The FTIR peaks at 2939 cm^−1^ and 2360 cm^−1^ indicate the presence of strong stretching amine salt (C–H) and carbon dioxide (CO_2_) respectively. Peak located at 1627 cm^−1^ and 1051 cm^−1^ denotes medium stretching conjugated alkene (C=C) and primary alcohol (C–O). Strong stretching nitro compound (N–O), alcohol (O–H), and alkyl aryl ether (C–O) are attributed at 1542 cm^−1^, 1396 cm^−1,^ and 1237 cm^−1^ respectively. In the FTIR spectra of the nanoparticle synthesized from Ginger (Fig. 4b), Zr, Garlic, Ginger (Fig. 5c), and Zr (Fig. 5d), a shift in the peaks was observed from 3266 to 3274, 2939 to 3941, 2360 to 2361, 1627 to 1637, 1626 and 1634, 1542 to 1534 and 1540, 1396 to 1388, 1051 to 1058, 1050 and 1048 cm^−1^. Some new peaks were also observed at 3343, 3291, and 1341 cm^−1^ corresponding to strong stretching alcohol (O–H), strong stretching alkyne (C–H), and medium bending phenol (O–H) respectively. Similar findings can be observed in the literature [[29], [30], [31], [32], [33]]. Table 1 shows the presence of bio-compounds in the synthesized nanoparticles.

**Table 1:** FTIR analysis data table of the synthesized nanofibers membrane.

Band (cm^−1^)	Functional class	Assignment	Vibration type
Garlic added ZrN nanoparticle			
3266	Carboxylic acid	O–H	Strong stretching
2939	Alkane	C–H	Medium stretching
2360	Carbon dioxide	O=C=O	Strong stretching
1627	Conjugated alkene	C=C	Medium stretching
1396	Alcohol	O–H	Medium bending
1237	Alkyl aryl ether	C–O	Strong stretching
1051	Primary alcohol	C–O	Strong stretching
Ginger added ZrN nanoparticle			
3291	Alkyne	C–H	Strong stretching
2360	Carbon dioxide	O=C=O	Strong stretching
1637	Conjugated alkene	C=C	Medium stretching
1396	Alcohol	O–H	Medium bending
1058	Primary alcohol	C–O	Strong stretching
Garlic and Ginger added ZrNnanoparticle			
3274	Carboxylic acid	O–H	Strong stretching
2941	Alkane	C–H	Medium stretching
2361	Carbon dioxide	O=C=O	Strong stretching
1626	Conjugated alkene	C=C	Medium stretching
1388	Aldehyde	C–H	Medium bending
1050	Primary alcohol	C–O	Strong stretching
ZrNnanoparticle			
3343	Alcohol	O–H	Strong stretching
2361	Carbon dioxide	O=C=O	Strong stretching
1634	Conjugated alkene	C=C	Medium stretching
1341	phenol	O–H	Medium bending
1048	Primary alcohol	C–O	Strong stretching

### 3.2 Field emission scanning electron microscope (FESEM) analyses

**Fig. 6:**
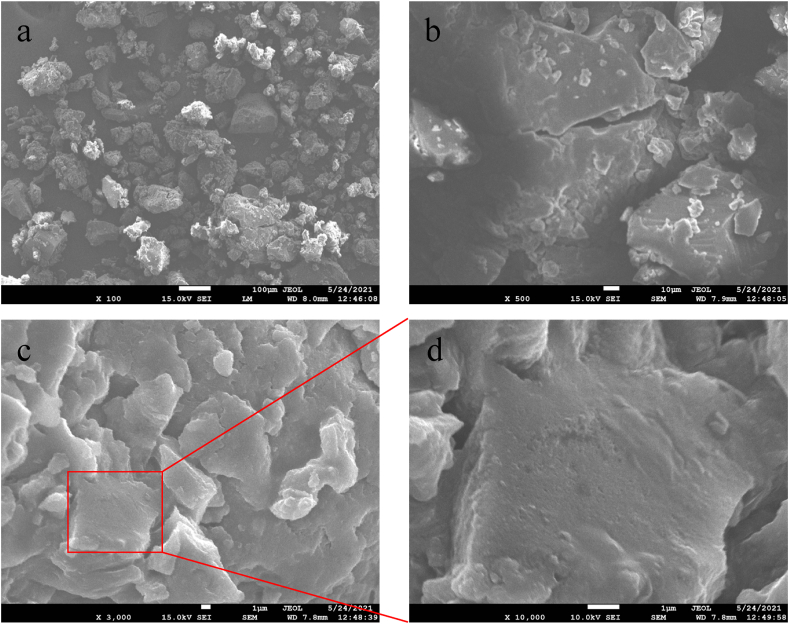
SEM images of the synthesizedZrN nanoparticles from Garlic at different magnifications.

**Fig. 7:**
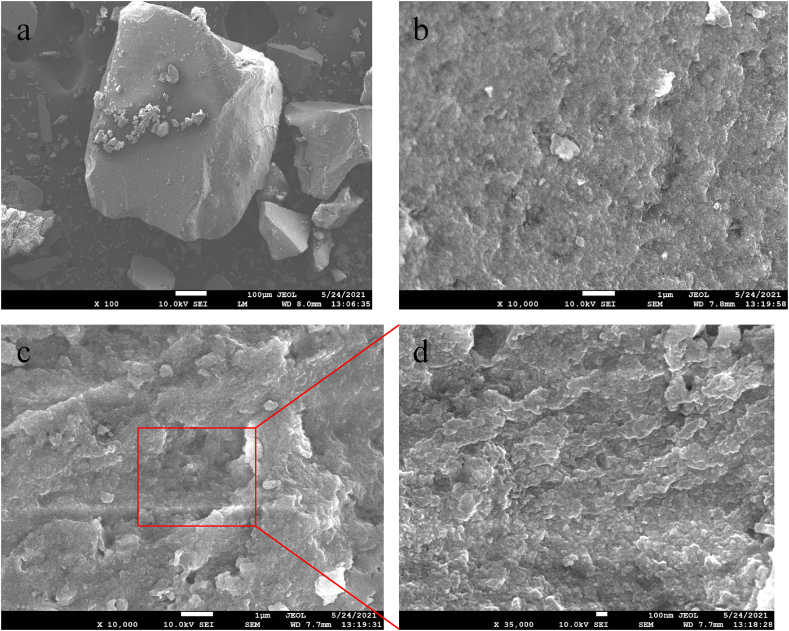
SEM images of the synthesized ZrNnanoparticles from Ginger at different magnifications.

**Fig. 8:**
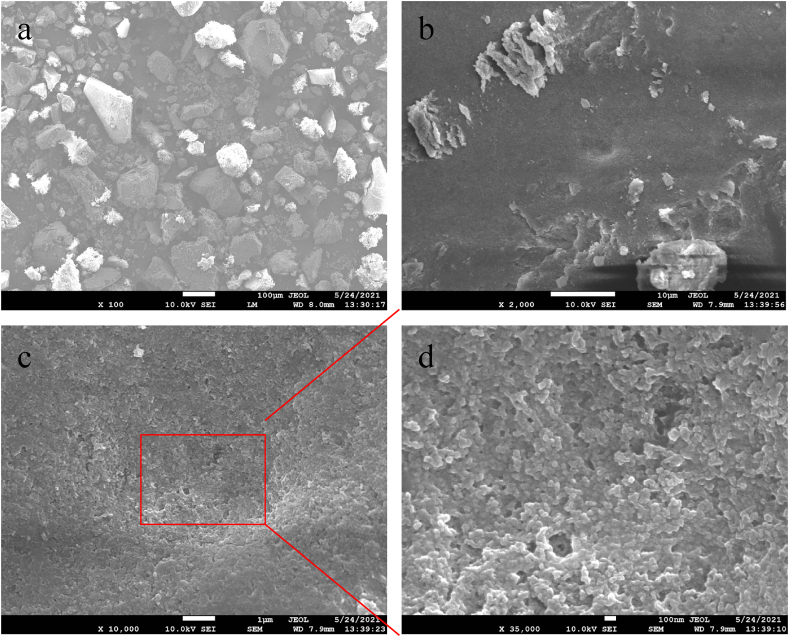
SEM images of the synthesized ZrNnanoparticles from Garlic and Ginger at different magnifications.

**Fig. 9:**
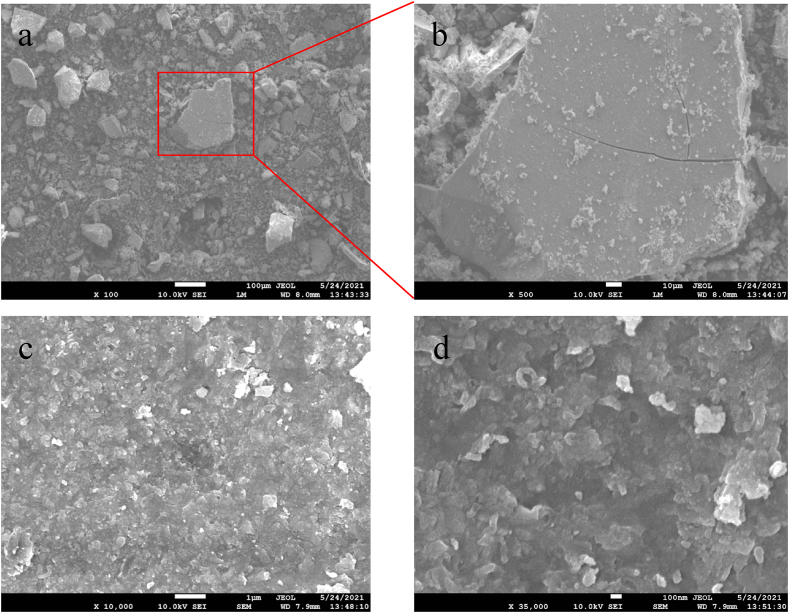
SEM images of the synthesized ZrNnanoparticles at different magnifications.

**Fig. 10:**
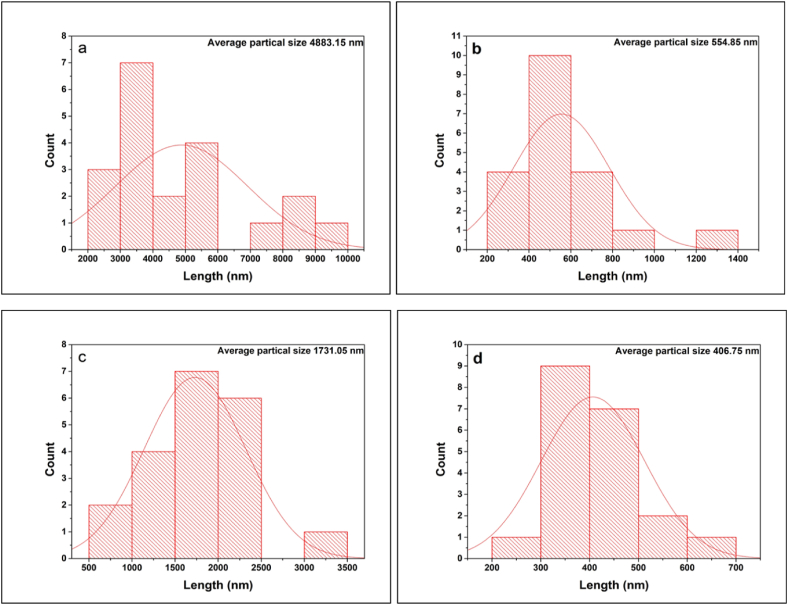
Particle size comparison of the synthesized (a) Garlic added ZrNnanoparticle, (b) Gingeradded ZrN nanoparticle, (c) Garlic and Ginger added ZrN nanoparticle(d) ZrN nanoparticle.

The morphology of the synthesized zirconium nanoparticles was detected by employing electron microscopy shown in Figs. Fig. 6, Fig. 7, Fig. 8, Fig. 9. The microphotography shows the dense packing of the particles and their uniform distribution which confirms the stability of the synthesized nanoparticles. The microstructure of the synthesized nanoparticles revealed that the as-synthesized nanoparticles are spherical, triangular, and irregular in shape [34,35]. The synthesized nanoparticles did not show any aggregation. Interactions between the various phytochemical molecules bound to the nanoparticles were indicated by FESEM images. Fig. 10 shows the size distribution plot of the nanoparticles. The particle size distribution curve confirmed the mean diameter of the synthesized nanoparticles. The average diameter of the zirconium nanoparticles synthesized from Garlic was found to be 4883 nm, Ginger 554 nm, Zr, Garlic and Ginger 1731 nm, and Zr 406 nm respectively.

### 3.3 Energy Dispersive X-Ray Spectroscopy (EDX) analysis

**Fig. 11:**
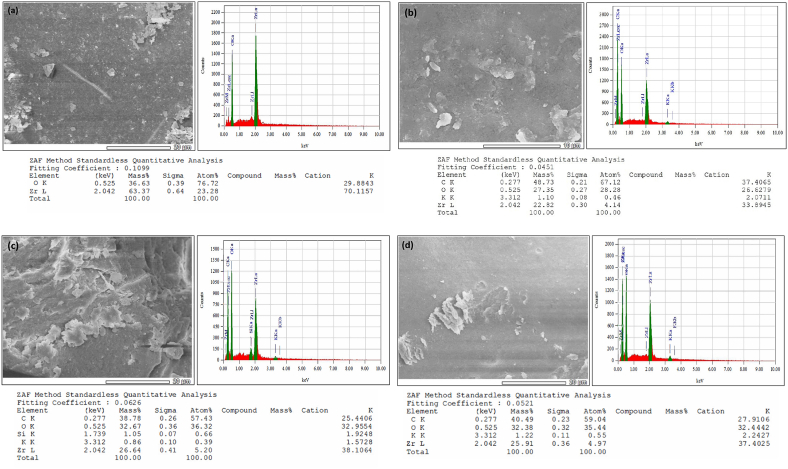
EDX analysis of the synthesized (a) Garlic added ZrNnanoparticle, (b) Ginger added ZrN nanoparticle, (c) Garlic and Ginger added ZrN nanoparticle (d) ZrN nanoparticle.

The elemental composition of the synthesized zirconium nanoparticles was analyzed by EDX shown in Fig. 11. The figures clearly depict the homogenous dispersion of zirconium in the synthesized nanoparticles. A notable number of peaks associated with O, K, C, Si, and Zr were observed and confirmed the production of zirconium nanoparticles. No impurities were observed in the obtained samples. From the figure, varying peaks are observed and perceived elemental composition. As the existence of O, K, C, Si, and Zr were verified by the appearance of the peaks, it can be said that the fabrication of the nanoparticles has been accordingly confirmed by the EDX. The percentages of all the elements were seen from the EDX results. In conformity with the available results, the existence of compounds in Garlic, Ginger, and ZrN within the construction of nanoparticles was verified [[36], [37], [38]].

### 3.4 4XRD analysis

**Fig. 12:**
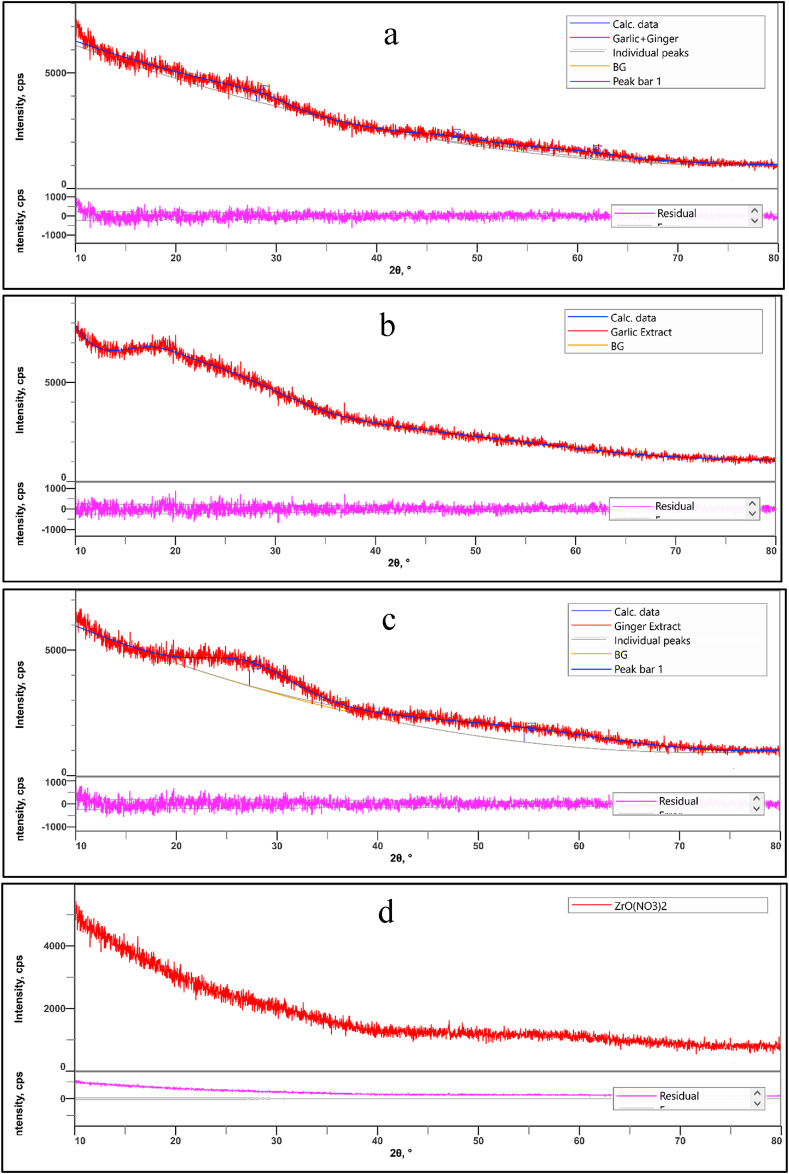
XRD analysis of the synthesized (a) Garlic added ZrNnanoparticle, (b) Ginger added ZrN nanoparticle, (c) Garlic and Ginger added ZrN nanoparticle (d) ZrN nanoparticle.

The formation of Zr nanoparticles from garlic, ginger, and ZrN is confirmed by the XRD pattern. Fig. 12 shows the XRD analysis of the synthesized Zr nanoparticles. The purity of the nanoparticles is indicated by the XRD spectra. The XRD pattern of Zr nanoparticles synthesized from the extract of garlic, ginger and shows a characteristic peak at 2θ values at 27.26°, 28.01°, 47.23°, 54.54°, and 61.31° planes. The crystalline size of the synthesized Zr nanoparticles varied. Previous literature shows similar results [[39], [40], [41]].

### 3.5 Antibacterial analysis

**Fig. 13:**
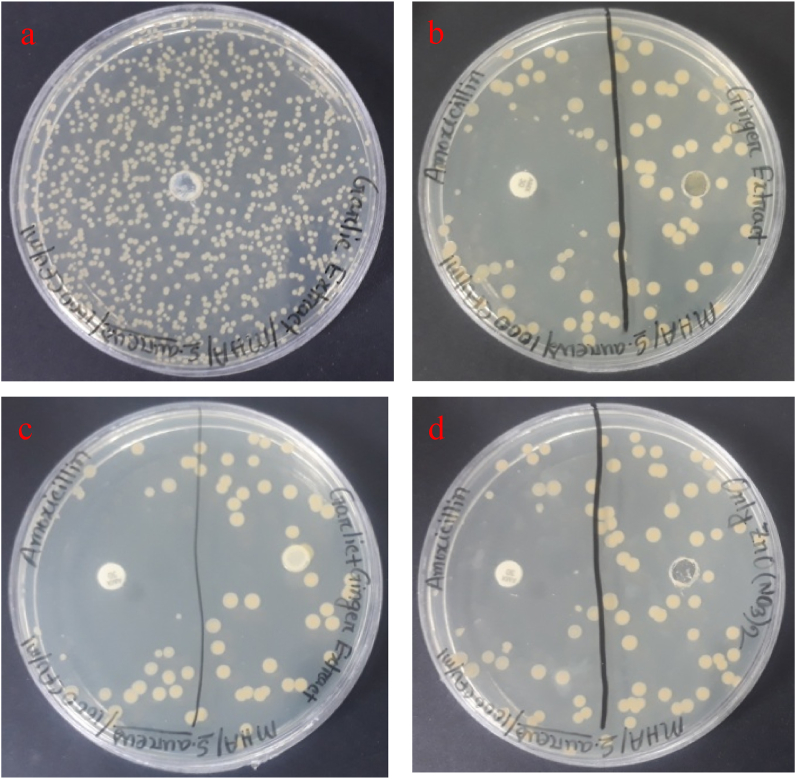
Antibacterial analysis of the synthesized nanoparticles synthesized with the help of (a) Garlic added Zr Nnanoparticle, (b) Ginger added ZrN nanoparticle, (c) Garlic and Ginger added ZrN nanoparticle (d) ZrN nanoparticle.

**Fig. 14:**
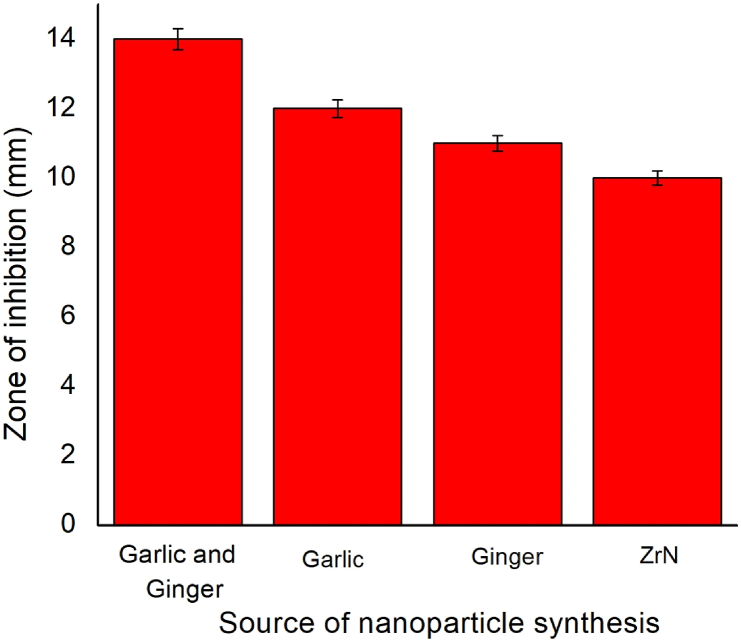
Bar diagram of in-vitro antibacterial studies of the synthesized nanoparticles synthesized with the help of different sources.

The present study shows the antibacterial activity of the zirconium nanoparticles synthesized from Garlic, Ginger, ZrN, Garlic and Ginger, and ZrN against *S. aureus using* the disc diffusion method. The nanoparticles synthesized from garlic with ginger extract show 14 ± 0.3 mm, garlic shows 12 ± 0.25 mm, ginger shows 11 ± 0.23 mm and ZrN shows 10 ± 0.2 mm inhibition against the bacteria. A similar observation can be found in the literature [[42], [43], [44]]. The bacterial inhibition is due to the release of diffusible inhibitory compounds from the zirconium nanoparticles. Direct contact of the nanoparticles by adhesion onto the surface of the cell wall and penetration to the microorganism and ion-mediated killing may be another reason. Other nanoparticles did not show any inhibition zone against the bacterial strain. The inhibition of the nanoparticles against the bacteria can be seen in Figs. 13 and 14. In general, extraction of the plant through nanoparticles can enhance the antimicrobial and biomedical applications but the selection of plant extracts depends on the health issues by which NPs can be used [[45], [46], [47]]. The results confirm that the synthesized nanoparticles can be successfully used as a dental implant for improved antibacterial efficiency.

### 3.6 Zr in dental implant applications and prospect

#### 3.6.1 Anti-biofilm characteristics

**Fig. 15:**
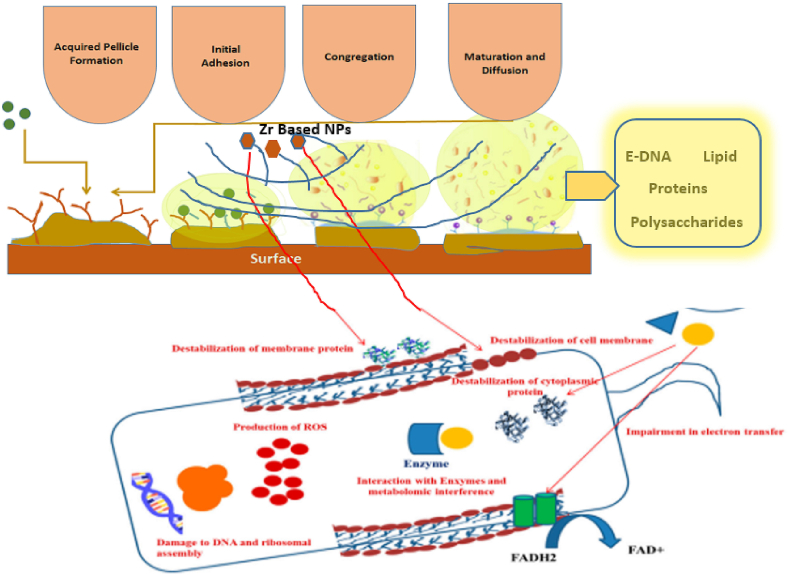
Biofilm formation in oral implant and anti-biofilm protection [Some portion of this figure adapted from ref. 50].

In general, the formation of dental biofilm (or dental plaque) involves several steps, beginning with the formation of the acquired enamel pellicle, followed by the initial adhesion of planktonic bacteria to the pellicle layer via binding sites, the subsequent maturation of the bacterial biofilm, and finally, the dispersion of the biofilm with cell/cluster detachment [48]. The biofilm formation process is illustrated in Fig. 15.

Biofilm production on oral implants can promote peri-implant tissue inflammation, putting the long-term effect of Osseointegrated implants in jeopardy. The use of a zirconium nitride top coat appears to be a promising surface modification approach for reducing bacterial attachment on implant surfaces and, as a result, potentially reducing implant infection caused by S. epidermidis biofilm formation [49]. The rapid advancement of zirconium-based nanotechnology has led to the creation of innovative nanoparticles as biocidal agents that can be easily integrated into biomaterials to inhibit microbial cell colonization or directly contact the pathogen by breaking through the biofilm matrix [50]. The antibiofilm efficacy of this nanotechnology is achieved by the creation of oxidative and nitride stressors and gene expression changes. The success of microorganism-assisted nanoparticle manufacturing cleared the way for such medicinal techniques, and it was considered to be more acceptable because of its “greener” approach by incorporating ginger and garlic extract.

#### 3.6.2 Formation of bio-integration

**Fig. 16:**
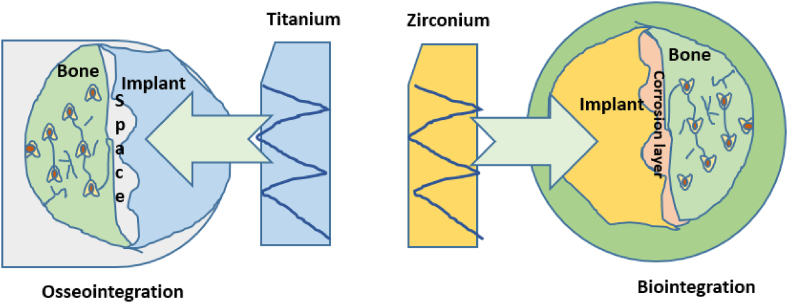
Osseointegration and bio-integration process.

For zirconia-based ceramic implant materials, there is no intervening gap between the ceramic and the bone. Rather, there is a connection between the implant and the bone. Bio-integration is the term for this state at the interface [51]. Bio-integration is assumed to necessitate a chemical breakdown of the ceramic implant that promotes bone development and allows it to merge with the surrounding bone, which contains a large amount of ceramic. The benefits of bio-integration over osseointegration, particularly, in the long run, are unknown. The clinical ankylosis of natural teeth is identical at both interfaces. Metallic implants covered with a ceramic promote a bio-integrated interface at first, but the interface's long-term stability is less evident because coatings erode with time. Osteointegration occurs when a Titanium alloy and supporting bone are in close proximity with no intervening fibrous tissue or collagen, implying that there is some space between the bone and the titanium alloy implantation [52]. The Osteointegration challenge in the case of titanium alloy can be mitigated by hybridization of titanium alloy with zirconium-based nanoparticles by suitable surface modification or power metallurgy technique in the future. The concept of osseointegration and bio-integration process is depicted in Fig. 16.

### 3.7 Cytotoxic effect analysis

**Fig. 17:**
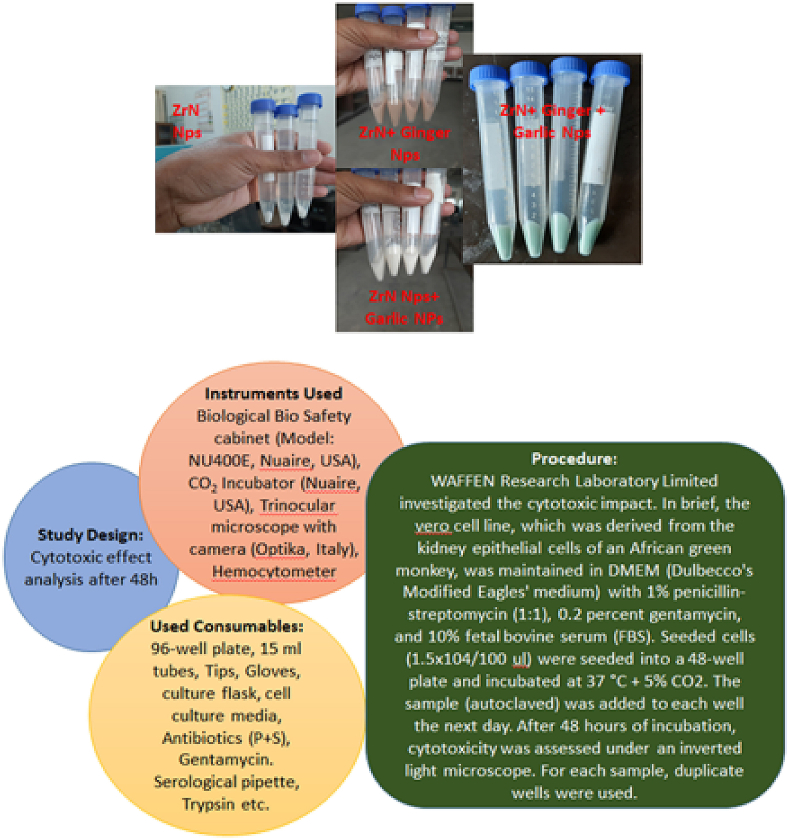
Cytotoxicity test design of nanoparticles.

Fig. 17 and Table 2 show the cytotoxicity test preparation and surviving cell results. When ZrN is infused with garlic and ginger, cell survival is 100%, but when simply ZrN is considered, cell survival is 85%. The majority of the cells, like the control groups, had original straight actin cables stretching from the perinuclear area to the cell periphery. These findings suggest that intracellular alterations in F-actin fibers happen before macroscopic morphological changes. Visualizing the actin cytoskeleton shape and organization validated the influence of exudates on cells and spreading. Actin microfilaments are required for cell shape and tight junction permeability. These results agree with results available in the literature [[53], [54], [55]].

**Table 2:** Cytotoxicity test results.

Sample ID	Survival of Cells	Remarks
	Vero	
Control	100%	Cell cytotoxicity was observed on Vero Cell line for ZrN + Garlic-Ginger extract not observed. But in case of ZrN, the survival cell is 85%.
ZrN + Garlic-Ginger extract	100%	
Only ZrN	85%	

### 3.8 Inclusion of garlic and ginger plant extract and their effects on dental health

Garlic extract can help to prevent dental cavities by increasing saliva production and inhibiting bacterial growth in the mouth. Alliin, allicin, SAC, DAS, allyl methyl disulfide, and ajoene are some of the bioactive chemicals found in garlic. Allicin, also known as diallyl-thiosulfinate, is a type of organo-sulfur secondary metabolite abundant in garlic and noted for its antibacterial characteristics. Resistance to allicin develops at a 1000-fold slower rate than resistance to antibiotics. Allicin works by inhibiting thiol group-containing enzymes, such as alcohol dehydrogenases and cysteine proteases, which are needed for pathogen tissue damage and survival. After tissue grinding, the alliinase (a cysteine sulfoxidelyase) enzyme secretes allicin from the garlic cloves. Other thiosulfinates, such as allin (allylthiosulfinate), ajoene, methyl allylthiosulfinates, and propenylallylthiosulfinates, are well-known quorum quenching molecules that inhibit bacterial growth due to their–S (O)–S- components, which interact with the sulfhydryl (SH) components of bacterial cell wall protein, The oral consumption of a few milligram concentrations of GE was proven to reduce both the gingival index (GI) and gingival bleeding index (GBI) in a randomized controlled clinical investigation, indicating that GE can relieve periodontal disorders as well. The twitching bacterial motility mechanism, which prevents bacterial colonization, is another mechanism that inhibits biofilm development. Furthermore, antibacterial activity is mostly due to the presence of phytochemicals such as tannins, flavonoids, and alkaloids in garlic extraction. Garlic extraction bulbs can be used to effectively treat periodontal and dental caries infections.

Garlic extract has a strong antifungal effect and inhibits the production of mycotoxins. The major component among multiple bioactive chemicals responsible for suppressing fungal growth was thought to be allicin (diallylthiosulphate). When garlic is sliced or crushed, the enzyme alliinase transforms alliin to allicin. Allicin has the ability to cross the membrane and bind to sulfur-containing chemical groups in proteins. As a result, glutathione is oxidized, causing microbial apoptosis to be activated. Garlic extraction has an antifungal effect due to the inhibitory impact of allicin (the active component of garlic) on thiol enzymes.

Garlic is high in phytochemicals, particularly allicin, which has antimicrobial properties. Allicin's principal active component, allyl methyl sulfide, interacts with viral phospholipids and amino acids involved in infection, preventing them from attaching to the host cell by denaturing them. These observations are also reported in the literature [[56], [57], [58], [59]]. Garlic extraction, as a natural chemical agent, can speed up the antioxidant, anticancer, and anti-inflammatory effects of dental implants [60].

Plant active principles are known as phytonutrients. They have biological relevance, despite the fact that they are not recognized as essential nutrients produced by plants. Ginger is a well-known source of these compounds, which have been linked to a variety of health benefits. Phenolic compounds (gingerol and shogaol), sesquiterpene hydrocarbons, and oleoresins are the principal active ingredients in ginger.10% ethanolic ginger extract has antibacterial action against oral pathogens that are resistant to commonly used antibiotics. Another study found that ginger extract at a dosage of 20 mg/ml is effective against *Pseudomonas aeruginosa*, and that the antibacterial activity was proportional to the extract concentration. Tumorigenesis is a multi-step process influenced by genetic and environmental variables. By activating apoptosis, upregulating tumor suppressor genes, and inhibiting angiogenic factors (vascular endothelial growth factor), ginger can help to prevent tumor growth and proliferation. Ginger is high in antioxidants, which work by scavenging free radicals such as superoxide anion, hydroperoxide, and hydroxyl. It also suppresses NO generation and prevents lipid peroxidation (LPO). Because of the inclusion of an unsaturated ketone moiety in their structures, compounds like 6 dehydroshogaol, 6-shogaol, and 1-dehydro-6-gingerdione have been implicated as powerful antioxidants. The use of systemic ginger extract can increase salivation rates. Salivation may be increased as a result of a direct parasympathomimetics action on post-synaptic M3 receptors as well as a putative restrictive effect on presynaptic muscarinic autoreceptors. Ginger's anti-inflammatory efficacy has been attributed to prostaglandin and leukotriene suppression, as well as dual inhibition of eicosanoid formation, according to studies on rheumatism and musculoskeletal illnesses. In dentistry, ginger's antibacterial, antifungal, antineoplastic, antioxidant, and anti-inflammatory properties are beneficial when combined with ZrN nanoparticles. These observations are reported in the literature [60,61]. Although ginger can be used to treat a variety of oral ailments, further clinical research is needed to better understand its benefits.

## 4 Conclusions

Garlic, ginger, and ZrN were used to successfully biosynthesize zirconium nanoparticles in a greener, safer, more eco-friendly, less expensive, and faster method. FTIR, FESEM, EDX, XRD, and antibacterial studies were used to characterize the produced nanoparticles. Depending on the natural elements used, the synthesized nanoparticles were spherical, triangular, and irregular in shape, with varying sizes. The existence of various bioactive chemicals in the produced nanoparticles was confirmed by IR spectra. All of the synthesized nanoparticles showed potential antimicrobial activity against *S. aureus*. Bacterial adhesion and biofilm formation have been shown to be inhibited by ZrNPs, and thickening the native ZrNPs coating may improve the corrosion barrier effect. The survival of cells, biointegration, and improved osseointegrationcapacities are some of the key findings of this study, both conceptually and experimentally. Overall, the findings indicate that nanoparticles have a promising future in dental implant applications. Despite the good results, it's worth emphasizing that long-term clinical implications have yet to be fully examined, and the osseointegration potential of Zr-based implants is still a point of concern.

## Declarations

### Author contribution statement

Dr. Mohammad Asaduzzaman Chowdhury: Conceived and designed the experiments.

Nayem Hossain: Performed the experiments; wrote the paper.

Md. Golam Mostofa; Md. Riyad Mia; Md. Tushar: Performed the experiments.

Masud Rana: Analyzed and interpreted the data.

MD. Helal Hossain: Contributed reagents, materials, analysis tools or data.

### Funding statement

This research did not receive any specific grant from funding agencies in the public, commercial, or not-for-profit sectors.

### Data availability statement

Data included in article/supp. material/referenced in article.

## Additional information

No additional information is available for this paper.

## Declaration of competing interest

The authors declare no conflict of interest.

## References

[1] Prasad RaiDhirendra, NareshCharmode Om PrakashShrivastav, Saurabh R., Prasad, AshaMoghe Prashant D. Sarvalkar, Prasad Neeraj R. (2021). A review on concept of nanotechnology in veterinary medicine. ES Food & Agroforestry.

[2] Ehab F., Mohamed Farag, Said Hanan A., Amin Abeer S., Azab Ehab, Gobouri Adil A., Fouda Amr, El-Belely (2021). Green synthesis of zinc oxide nanoparticles (ZnO-NPs) using Arthrospiraplatensis (Class: cyanophyceae) and evaluation of their biomedical activities. Nanomaterials.

[3] Huston Matthew, DeBella Melissa, Bella MariaDi, Gupta Anisha (2021). Green synthesis of nanomaterials. Nanomaterials.

[4] Salem Salem S., Fouda Amr (2021). Green synthesis of metallic nanoparticles and their prospective biotechnological applications: an overview. Green trace element research.

[5] Chowdhury Mohammad Asaduzzaman, Hossain Nayem, Kchaou Mohamed, Nandee Rajib, Bengir Md, Shuvho Ahmed, Sultana Sadia (2021). Scope of eco-friendly nanoparticles for anti-microbial activity. Current Research in Green and Sustainable Chemistry.

[6] Chowdhury Mohammad Asaduzzaman, Hossain Nayem, AbulKashem Mohammod, Shahid MdAbdus, Alam Ashraful (2020). Effects of nanoparticles on viral infection—a review. Nano.

[7] Hossain Nayem, Chowdhury Mohammad Asaduzzaman, Sultana Sadia, Nandee Rajib (2022). Scope of 2D materials for immune response-a review. Results in Engineering.

[8] Kulkarni Amritha G., De Britto Savitha, Jogaiah Sudisha (2021).

[9] Kumar D. Dinesh, SaravananKaliaraj Gobi (2018). Multifunctional zirconium nitride/copper multilayer coatings on medical grade 316L SS and titanium substrates for biomedical applications. J. Mech. Behav. Biomed. Mater..

[10] Rohr Nadja, Märtin Sabrina, Fischer Jens (2021). Fracture load of zirconia implant supported CAD/CAM resin crowns and mechanical properties of restorative material and cement. Journal of Prosthodontic Research.

[11] Hatim Alqurashi, Khurshid Zohaib, Syed AzeemUlYaqin, Rashid Habib Syed, Rokaya Dinesh, Sohail Zafar Muhammad (2021). Polyetherketoneketone (PEKK): an emerging biomaterial for oral implants and dental prostheses. J. Adv. Res..

[12] Ghomi Ahmad Reza Golnaraghi, Mohammadi-Khanaposhti Mohammad, Vahidi Hossein, Kobarfard Farzad, Shah Reza MahdiehAmeri, Barabadi Hamed (2019). Fungus-mediated extracellular biosynthesis and characterization of zirconium nanoparticles using standard penicillium species and their preliminary bactericidal potential: a novel green approach to nanoparticle synthesis. Iran. J. Pharm. Res. (IJPR): IJPR.

[13] Ghomi Ahmad Reza Golnaraghi, Mohammadi-Khanaposhti Mohammad, HosseinVahidi FarzadKobarfard, Shah Reza MahdiehAmeri, HamedBarabadi (2019). Fungus-mediated extracellular biosynthesis and characterization of zirconium nanoparticles using standard penicillium species and their preliminary bactericidal potential: a novel green approach to nanoparticle synthesis. Iran. J. Pharm. Res. (IJPR): IJPR.

[14] Konappa Narasimhamurthy, Udayashankar Arakere C., Dhamodaran Nirmaladevi, Krishnamurthy Soumya, Jagannath Shubha, Uzma Fazilath, Pradeep ChamanahalliKyathegowda, Britto Savitha De, Chowdappa Srinivas, Jogaiah Sudisha (2021). Ameliorated antibacterial and antioxidant properties by trichodermaharzianum mediated green synthesis of silver nanoparticles. Biomolecules.

[15] Nayak Sreenivasa, Bhat Meghashyama P., Udayashankar A.C., Lakshmeesha T.R., Geetha Nagaraja, Jogaiah Sudisha (2020). Biosynthesis and characterization of Dilleniaindica-mediated silver nanoparticles and their biological activity. Appl. Organomet. Chem..

[16] SudishaJogaiah MahanteshKurjogi, MostafaAbdelrahman NagabhushanaHanumanthappa, Son Phan Tran Lam (2019). Ganodermaapplanatum-mediated green synthesis of silver nanoparticles: structural characterization, and in vitro and in vivo biomedical and agrochemical properties. Arab. J. Chem..

[17] Ahmad Hilal, Krishnan Venugopal, KalyanaramanRajagopal Savitha De Britto, BoregowdaNandini, HosurGnanaprakashPushpalatha, NarasimhamurthyKonappa Arakere C., Udayashankar, NagarajaGeetha, SudishaJogaiah (2020). Green synthesis and characterization of zinc oxide nanoparticles using Eucalyptus globules and their fungicidal ability against pathogenic fungi of apple orchards. Biomolecules.

[18] Gao Yang, Ozel Mustafa Z., Tom Dugmore, AllynSulaeman, Matharu Avtar S. (2021). A biorefinery strategy for spent industrial ginger waste. J. Hazard Mater..

[19] El-Borm, Hend T., Gobara Marwa S., Badawy Gamal M. (2021). Ginger extract attenuates labetalol induced apoptosis, DNA damage, histological and ultrastructural changes in the heart of rat fetuses. Saudi Journal of Green Sciences.

[20] Hamza Alaaeldin Ahmed, Gehan Hussein Heeba SalsabilHamza, Ali Abdalla, Amin Amr (2021). Standardized extract of ginger ameliorates liver cancer by reducing proliferation and inducing apoptosis through inhibition oxidative stress/inflammation pathway. Biomed. Pharmacother..

[21] Yan Jing-Kun, Wang Chun, Yu Yun-Bo, Wu Li-Xia, Chen Ting-Ting, Wang Zi-Wei (2021). Physicochemical characteristics and in vitro green activities of polysaccharides derived from raw garlic (Allium sativum L.) bulbs via three-phase partitioning combined with gradient ethanol precipitation method. Food Chem..

[22] De Greef Danielle, Barton Emily M., Sandberg Elise N., Croley Courtney R., Pumarol Joshua, Wong Tin Lok, Das Niranjan, Bishayee Anupam (2021).

[23] Moreira André, Madeira Sara, Buciumeanu Mihaela, Fialho Joana, Carvalho Angela, Silva Filipe, Monteiro Fernando J., Caramês João (2022). Design and surface characterization of micropatterned silica coatings for zirconia dental implants. J. Mech. Behav. Biomed. Mater..

[24] Chopra Divya, AnjanaJayasree TianqiGuo, Gulati Karan, SašoIvanovski (2021). Advancing dental implants: bioactive and therapeutic modifications of zirconia. Bioact. Mater..

[25] Qu Yinying, Liu Lin (2021). Zirconia materials for dental implants: a literature review. Frontiers in Dental Medicine.

[26] Vasylyev M.A., Mordyuk B.N., Sidorenko S.I., Voloshko S.M., Burmak A.P., Kruhlov I.O., Zakiev V.I. (2019). Characterization of ZrN coating low-temperature deposited on the preliminary Ar+ ions treated 2024 Al-alloy. Surf. Coating. Technol..

[27] Raura Natasha, AnirudhGarg ArpitArora, Roma M. (2020). Nanoparticle technology and its implications in endodontics: a review. Biomater. Res..

[28] Chau Tan Phat, Veeraragavan GeethaRoyapuram, Narayanan Mathiyazhagan, Chinnathambi Arunachalam, Ali Alharbi Sulaiman, Subramani Baskaran, Brindhadevi Kathirvel, Pimpimon Tipsukon, Pikulkaew Surachai (2022). Green synthesis of zirconium nanoparticles using pomegranate peel extract and their antimicrobial and antioxidant potency. Environ. Res..

[29] Varghese Bincicil Annie, Nair ReshmaVijayakumariRaveendran, Jude Shintu, Varma Karthik, Amalraj Augustine, Kuttappan Sasikumar (2021). Green synthesis of gold nanoparticles using Kaempferiaparviflora rhizome extract and their characterization and application as an antimicrobial, antioxidant and catalytic degradation agent. J. Taiwan Inst. Chem. Eng..

[30] Kolahalam Lalitha A., Prasad K.R.S., Murali Krishna P., Supraja N. (2021). Saussurealappa plant rhizome extract-based zinc oxide nanoparticles: synthesis, characterization and its antibacterial, antifungal activities and cytotoxic studies against Chinese Hamster Ovary (CHO) cell lines. Heliyon.

[31] AlSalhi Mohamad S., Elangovan Kannan, Ranjitsingh Amirtham Jacob A., Murali P., Devanesan Sandhanasamy (2019). Synthesis of silver nanoparticles using plant derived 4-N-methyl benzoicacid and evaluation of antimicrobial, antioxidant and antitumor activity. Saudi Journal of Green Sciences.

[32] Muthukumar Harshiny, Palanirajan Santosh Kumar, Kumar Shanmugam Manoj, Gummadi Sathyanarayana N. (2020). Plant extract mediated synthesis enhanced the functional properties ofsilver ferrite nanoparticles over chemical mediated synthesis. Biotechnology Reports.

[33] Manik U.P., AmolNande, Raut Swati, Dhoble S.J. (2020). Green synthesis of silver nanoparticles using plant leaf extraction ofArtocarpusheterophylus and Azadirachtaindica. Results in Materials.

[34] Dhar SajibAninda, Chowdhury RashedulAlam, Das Shaon, Khalid Nahian Md, DipaIslam Md, Gafur Abdul (2021). Plant-mediated green synthesis and characterization of silver nanoparticles using Phyllanthusemblica fruit extract. Mater. Today Proc..

[35] Kumar Deepak, ShefaliArora Abdullah, Danish Mohd (2019). Plant based synthesis of silver nanoparticles from ougeiniaoojeinensis leaves extract and their membrane stabilizing, antioxidant and antimicrobial activities. Mater. Today Proc..

[36] Rahmani Reyhaneh, Gharanfoli Mohsen, Gholamin Mehran, Darroudi Majid, Chamani Jamshidkhan, Sadri Kayvan, Hashemzadeh Alireza (2020). Plant-mediated synthesis of superparamagnetic iron oxide nanoparticles(SPIONs) using aloe vera and flaxseed extracts and evaluation of theircellular toxicities. Ceram. Int..

[37] Veisi Hojat, Tamoradi Taiebeh, Karmakar Bikash, Mohammadi Pourya, Hemmati Saba (2019). Insitu biogenic synthesis of Pd nanoparticles over reduced grapheneoxideby using a plant extract (Thymbraspicata) and its catalytic evaluationtowardscyanation of aryl halides. Mater. Sci. Eng. C.

[38] Thakur Atul, Sharma Nidhi, Bhatti Manpreet, Sharma Monica, Trukhanov Alex V., Trukhanov Sergei V., Panina Larissa V., Astapovich Ksenia A., Thakur Preeti (2020). Synthesis ofbarium ferrite nanoparticles using rhizome extract ofAcorusCalamus: characterization and its efficacy against differentplantphytopathogenic fungi. Nano-Structures & Nano-Objects.

[39] UllahMirza Azar, Kareem Abdul, Nami Shahab A.A., Ahmad Bhat Shahnawaz, Mohammad Abdulrahman, Nishat Nahid (2019). Maluspumila and Juglenregia plant species mediated zinc oxidenanoparticles: synthesis, spectral characterization, antioxidant andantibacterial studies. Microb. Pathog..

[40] Meydan Ismet, Seckin Hamdullah, Bekmezci Muhammed, Sen Fatih, Tugba Gur (2022). Green synthesis, characterization and bioactivity of biogenic zincoxide nanoparticles. Environ. Res..

[41] Falih Alaa, Ahmed Naser M., Rashid Marzaini (2022). Green synthesis of zinc oxide nanoparticles by fresh and dry alhagi plant. Mater. Today Proc..

[42] Kolahalam Lalitha A., Prasad K.R.S., Murali Krishna P., Supraja N. (2021). Saussurealappa plant rhizome extract-based zinc oxide nanoparticles: synthesis, characterization and its antibacterial, antifungal activities and cytotoxic studies against Chinese Hamster Ovary (CHO) cell lines. Heliyon.

[43] Anandalakshmi K., Venugobal J., Ramasamy V. (2016). Characterization of silver nanoparticles by green synthesis method using Pedalium murex leaf extract and their antibacterial activity. Appl. Nanosci..

[44] Violet Mary J., Pragathiswaran C., Anusuya N. (2021). Photocatalytic, degradation, sensing of Pb^2+^ using titanium nanoparticles synthesized via plant extract of Cissusquadrangularis: in-vitroanalysis of microbial and anti-cancer activities. J. Mol. Struct..

[45] Chowdhury Mohammad Asaduzzaman, Hossain Nayem, Kchaou Mohamed, Nandee Rajib, Ahmed Shuvho MdBengir, Sultana Sadia (2021). Scope of eco-friendly nanoparticles for anti-microbial activity. Current Research in Green and Sustainable Chemistry.

[46] Chowdhury Mohammad Asaduzzaman, Hossain Nayem, AbulKashem Mohammod, Shahid MdAbdus, Alam Ashraful (2020). Effects of nanoparticles on viral infection—a review. Nano.

[47] Hossain Nayem, Chowdhury Mohammad Asaduzzaman, Sultana Sadia, Nandee Rajib (2022). Scope of 2D materials for immune response-a review. Results in Engineering.

[48] Abebe G.M. (2021). Oral biofilm and its impact on oral health, psychological and social inter-action. Int J Oral Dent Health.

[49] Pilz Magdalena, Staats Kevin, Tobudic Selma, Assadian Ojan, Presterl Elisabeth, Windhager Reinhard, Johannes Holinka (2019). Zirconium nitride coating reduced Staphylococcus epidermidis biofilm formation on orthopaedic implant surfaces: an in vitro study. Clin. Orthop. Relat. Res..

[50] Nag Moupriya, Lahiri Dibyajit, Sarkar Tanmay, Ghosh Sujay, Dey Ankita, AtanEdinur Hisham, Pati Siddhartha, Ray Rina Rani (2021). Microbial fabrication of nanomaterial and its role in disintegration of exopolymeric matrices of biofilm. Front. Chem..

[51] Bosshardt Dieter D., Chappuis Vivianne, Daniel Buser (2017). Osseointegration of titanium, titanium alloy and zirconia dental implants: current knowledge and open questions. Periodontol. 2000.

[52] Victor SunitaPrem, Pillai C.K.S., Sharma Chandra P. (2020). Biointegration of Medical Implant Materials.

[53] Van Cruchten Steven, Van Den Broeck Wim (2002). Morphological and biochemical aspects of apoptosis, oncosis and necrosis. Anat. Histol. Embryol..

[54] Stan Miruna-Silvia, Memet Indira, Fratila Cornel, Roman Ioan, Dinischiotu Anca, Elzbieta Krasicka-Cydzik (2015). Effects of titanium‐based nanotube films on osteoblast behavior in vitro. J. Biomed. Mater. Res..

[55] Shin Hyeongsoon, Ko Hyunjung, Kim Miri (2016). Cytotoxicity and biocompatibility of Zirconia (Y-TZP) posts with various dental cements. Restorative dentistry &endodontics.

[56] Sasi Minnu, Kumar Sandeep, Kumar Manoj, Thapa Sandhya, Prajapati Uma, Tak Yamini, Changan Sushil (2021). Garlic (allium sativum L.) bioactives and its role in alleviating oral pathologies. Antioxidants.

[57] Hutomo Suryani, UtamiPutri Denise, Welviyanda BeatricChindy, Susilowati Heni (2021). Inhibition effect of garlic (allium sativum) extract on Streptococcus sanguinis Biofilm Formation involving bacterial motility mechanism. Malays. J. Med. Health Sci.

[58] Papu Singh, Singh Jaivir, Singh Sweta, Singh B.R. (2014). Medicinal values of garlic (Allium sativum L.) in human life: an overview. Greener Journal of Agricultural Sciences.

[59] Rashmi K.J., Tiwari Ritu (2016). Pharmacotherapeutic properties of ginger and its use in diseases of the oral cavity: a narrative review. J. Adv. Oral Res..

[60] Jung Hyo Won, Yoon Cheol-Ho, Kwon Moo Park, Han HyungSoo, Park Yong-Ki (2009). Hexane fraction of ZingiberisRhizomaCrudus extract inhibits the production of nitric oxide and proinflammatory cytokines in LPS-stimulated BV2 microglial cells via the NF-kappaB pathway. Food Chem. Toxicol..

[61] Park Miri, Bae Jungdon, Lee Dae-Sil (2008). Antibacterial activity of [10]‐gingerol and [12]‐gingerol isolated from ginger rhizome against periodontal bacteria. Phytother Res.: An International Journal Devoted to Pharmacological and Toxicological Evaluation of Natural Product Derivatives.

